# Desert Island Papers

**DOI:** 10.1177/23982128231199006

**Published:** 2023-09-19

**Authors:** Kate Baker, Jeffrey W. Dalley

**Affiliations:** 1 MRC Cognition and Brain Sciences Unit, University of Cambridge, Cambridge, UK; 2 Department of Medical Genetics, University of Cambridge, Cambridge, UK; 3 Department of Psychology, University of Cambridge, Cambridge, UK; 4 Department of Psychiatry, Herchel Smith Building for Brain and Mind Sciences, Cambridge, UK

**Keywords:** Scientific writing, science communication, career development, neuroscience

## Abstract

This article presents edited highlights from a special session at the BNA International Festival of Neuroscience held in Brighton in April 2023. The session involved Desert Island Disc–style interviews between early career researchers and established investigators, discussing papers that influenced their neuroscience careers.

## Introduction

*The Voyage* – a celebration of reading and writing journal articles, as critical processes for neuroscientific progress, team science, career development and personal satisfaction.

*The Island* – the Skyline room of the Brighton Centre, with uninterrupted views of the English Channel, on the closing day of the Festival.

*The Castaways* – three influential neuroscientists with a love of reading and writing papers.

*The Interviewers* – three early career research leaders with passion for conducting and communicating neuroscience.

## Shipwreck one

Castaway **Dr Mike Ashby (MA)**, Senior Lecturer at the School of Physiology, Pharmacology and Neuroscience, University of Bristol; interviewed by **Dr Faye McLeod (FM)**, Epilepsy Research UK Emerging Leader Fellow at the Biosciences Institute, Newcastle University.


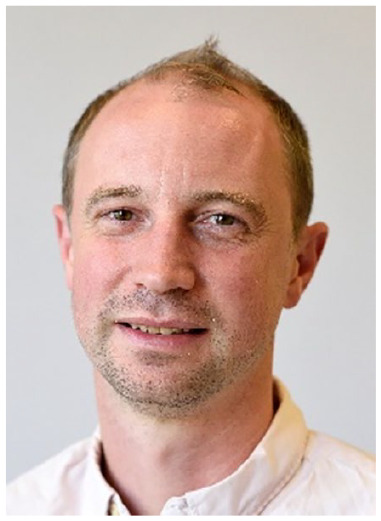
 
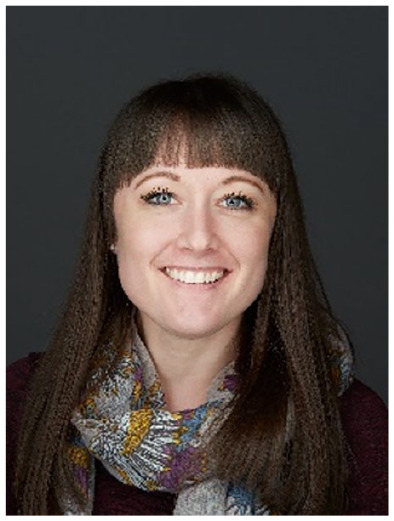

FM: It’s a pleasure to be interviewing you, Mike. I have followed your research from my time as a PhD student to the point of becoming an independent researcher. Can you tell us about a paper that inspired you early in your career?

MA: The paper that stands out is one that I read when I first stepped into neuroscience as a postdoc. It was from Eve Marder’s Lab and David Schultz is the first author (Schulz et al., 2007). If Eve Marder writes something, I implore you to read it because it’s brilliant! Her lab works on neurons in crabs. Crabs have a very stereotyped nervous system, so one crab is the same as the other in terms of where the neurons are. They did amazing single-cell recordings in different crabs and then extracted RNA to figure out what those neurons were expressing. The amazing finding, which changed the way I thought about how neurons work, is that ion channel expression was different in the different cells, and yet they all have the same spiking pattern. It turned out to be the combination of the expression pattern that is important, not the individual genes. I think that’s really important now, because we’re in a phase of neuroscience where everyone’s trying to characterise different cells by their expression patterns, but the same cell can perform the same function with different expression patterns. So I think it’s a really insightful paper.

FM: I think there is a tendency to focus on ‘it has to be mammalian cells that we look at’, but the appreciation of a simplified model system is important, considering how signalling systems can be conserved or be different.

MA: I think at the heart of that, there is a strong message of don’t be too wedded to your own model. There’s a lot of value in studying things in different ways.

FM: So what was the first paper that you published, and how did you feel at the time?

MA: It was quite a while ago now, as you might imagine looking at me, but I remember it very well. It was the second or third year of my PhD, and I was very excited about trying to publish my work. We submitted it and the reviews pummelled it into the ground – I remember the feeling that someone had kicked me in the guts. But when I calmed down and spoke to my supervisor, we came up with experiments to address the reviewers’ comments, some of which were totally valid and fair. This opened-up another part of the study and added two or three new figures to the rewritten manuscript. We submitted it a year later to another journal and it flew (Ashby et al., 2002). Then I was totally euphoric because when you put the extra effort in and get your paper out, and people start to read it – that’s pretty cool.

FM: I love writing about the science I am doing. As you progress through your career, do you find that you get the same feelings about publishing, or how has that changed over your career?

MA: I think it has changed a little bit. I don’t feel the kick in the guts quite so hard because I expect it and you learn not to take it too personally. There’s a process of trying to do the best science, and review and publication is part of that. Criticism is something we should all be open to. Sometimes we don’t agree with it, but it’s the right thing to do to make the best science. And the extra joy I get out of it now, when we publish a paper and I’m the senior author, is from the benefit to the people who’ve done the legwork. So that’s a slightly different feeling, but it’s equally as good. I hope that the students and postdocs in my lab get some benefit from seeing the way I write. But it’s very notable that people have different styles and ways they write, so there’s always something to learn in that process.

FM: So the influence you have now is by sharing your experience and sharing the skills. Is there one paper you’ve published which you’re most proud of?

MA: There’s lots of papers that I look back on fondly, because it’s not just the paper but the process and the project. I am proud of the first paper I published after switching to neuroscience after my PhD – it was a validation that I’d done the right thing by jumping ship over to neuroscience. It was about trafficking of AMPA receptors in dendritic spines (Ashby et al., 2006). The consensus was that receptors moved around in vesicles and then slotted into the membrane at the synapse. We showed that lateral movement along the membrane was a big deal – the receptors were actually moving sites and sliding in rather than being delivered. I remember going to a meeting much like this conference and presenting the findings as a poster, and tons of people came along and asked questions. There were several themes that people asked about, so it became clear that we had to address these before we completed the study. I did those experiments as soon as I got back to Bristol, which became an important part of the paper. So it felt like there was community support for the paper, and there was also a strong collaboration with my PI. I was very pleased that I managed to publish a paper in neuroscience.

FM: Are you able to tell us about any publications you’re working on now?

MA: Embarrassingly, I’ve probably got five or six things on the go. There is one by Soraya Meftah, who is now in Edinburgh as a postdoc. She did some lovely work on a mouse model of dementia, doing really difficult single-cell recordings in vivo. The data are really beautiful. This was one of these projects where there are multiple labs involved, so the paper is going round everyone, and it has taken us too long really. But that’s something that you have to take care of, to keep the energy going. It’s top of my to-do list as soon as I get back from here. [Editorial addition: paper became available as a preprint one month later (Meftah et al., 2023).]

FM: What would be your desert island luxury to get you through the writing and publishing process?

MA: Sometimes publishing papers can give you a little knock, right? Because you do get criticism. So I think I’d like a source of continual support. I would take my family dog with me – Toby is unconditional in his support for me, all the time. So that would be a nice antidote to those moments of self-doubt or knocks that you get. Because in the end you do come out the other side.

## Shipwreck two

Castaway **Professor Sarah-Jayne Blakemore (SJB)**, Professor of Psychology and Cognitive Neuroscience at the Department of Psychology, University of Cambridge; interviewed by **Emma Soopramanien (ES)**, MSc student at UCL Institute of Cognitive Neuroscience, and BNA Committee Students and Early Career Representative.


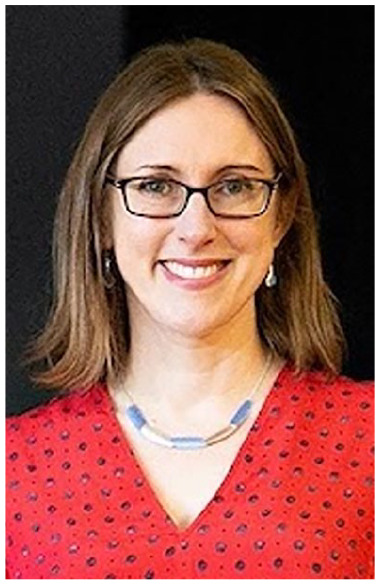
 
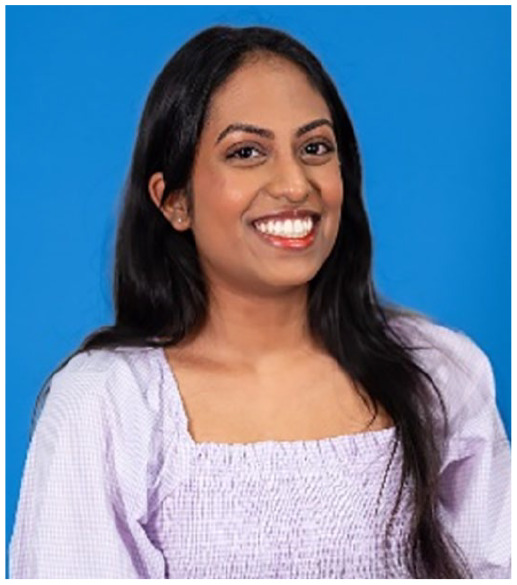

ES: Akin to Faye, I’ll start off in chronological order by asking you about your early career. Is there a paper that influenced your steps toward working in neuroscience?

SJB: The paper I have chosen is by Simon Baron-Cohen, Alan Leslie and Uta Frith called ‘Does the Autistic Child Have a Theory of Mind?’ (Baron-Cohen et al., 1985). It was published when I was 11, but I didn’t read it when I was 11. A few years later, a psychologist at Oxford called Peter Bryant (you might know his work on dyslexia) came to give a talk at my school. I went up to him afterwards and said ‘I’m really interested in developmental psychology. How can I find out more?’ and he had in his bag Uta Frith’s brand-new book called Autism (Frith, 1989). I don’t think he’d even read it, but he said ‘This is what you’ll be interested in’. So I read it and then I wrote to Uta and asked if I could do work experience with her. I went to work in her lab for a week in the summer after my GCSEs. At that point, I read her paper with Baron-Cohen and Leslie, and that really triggered my interest in experimental psychology. I learnt afterwards that this paper was radical at the time because until then autism was thought of as an emotional disorder, caused by cold parenting (by which people really meant cold mothering). That paper completely changed the way people thought about autism, as something to do with cognitive development and the brain. There’s a lot you could say about whether theory of mind is different in autism – there are massive individual differences – but the idea that autism results from diverse development of cognition was pioneered by Uta.

ES: That is incredible! The thing that stood out to me is how you read a paper at age 15 or 16. I was struggling to do that as an undergraduate. Just that level of concentration and how driven you were and passionate about the field. I think that’s admirable.

SJB: I really wasn’t very good at concentrating at the age of 15. I wasn’t doing very well at school, and I wasn’t that motivated by lessons. That work experience was the thing that showed me that academic work could be really exciting. It laid the foundations for my wanting to study psychology at university.

ES: And from that, I will pivot slightly and ask what was the first paper you published?

SJB: My first paper (I had to look this up) was called ‘How do We Predict the Sensory Consequences of our Actions? A functional imaging study’ (Blakemore et al., 1998a) and it was with Geraint Rees and Chris Frith. My PhD focused on how we predict the sensory consequences of our actions and it was an experimental investigation of Daniel Wolpert’s theory of forward prediction models in the brain. All you need to know about this paper is that it contains six participants, because back then, it was easy to publish neuroimaging papers, especially if you flagged it in the title. I used really basic analysis – just fixed effects, no random effects, no corrections for multiple comparisons across the whole brain. It might have been all noise, and there were no strong a priori hypotheses because there were no previous neuroimaging studies to base hypotheses on. It was about predicting auditory stimuli that you cause by pressing a button, versus hearing an externally generated sound. What this teaches you is that methods evolve very rapidly and never to be complacent about the methods that you’re using because probably in five or ten years’ time they will have moved on. Another point is about replication – I don’t think that anyone’s tried to replicate that study. If they managed to, I’d be very amazed and delighted! The paper was superseded by another we wrote on self-produced touch (Blakemore et al., 1998b). It also had fixed effects analysis and small sample size, but the results were hypothesised based on previous research and theoretical models, and we were looking for specific changes in activity when you produced touch as opposed to the same touch being externally produced. That study has been replicated, which is really important. Over the years, I’ve learned that doing slower science and focusing on quality over quantity, and doing your own replications even if it delays your paper by a year (or two or three), makes for a better paper, which will have more impact.

ES: That is a great segway into my next question, because you’re saying publication can take longer than initially planned. Could you tell me about a paper that has been difficult to publish for whatever reason?

SJB: Yes, we all have problems publishing some papers. It’s very random because it depends on what reviewer you happen to get. I wanted to focus here on something that was objectively challenging. I was involved in the Myriad study, led by William Kuyken and Mark Williams in Oxford (https://myriadproject.org/). The idea was that mindfulness had been shown to be beneficial for mental health in adults. Schools all over the world were rolling out mindfulness classes because ‘it’s really good for mental health’. But, there had only been a few small studies and no sufficiently powered trial of mindfulness interventions in schools. So that’s what we set out to do. We randomly divided around 90 schools into two groups – one group continued standard social-emotional learning lessons as normal, and the other group replaced some of these lessons with eight weeks of mindfulness. We hypothesised that mindfulness would improve children’s mental health, measured in a variety of ways. After eight years and a huge amount of work, we found that it did not – there were no significant differences between the two groups. That was disappointing, but it was also crucial because a well-powered study with a null result is more important than a smaller (underpowered) study that shows a positive impact of an intervention. The implications are that, as a universal intervention, mindfulness is probably not a good use of school resources. When we began this study, we published a protocol (Kuyken et al., 2017) and the Wellcome Trust publicised it (because this was funded by a Wellcome strategic award), and a few very high-impact journals got in touch saying they were interested in publishing the results. When we had null results, we immediately approached these journals, and they said ‘Oh no thanks’. They didn’t really come up with good reasons, but it seemed likely that it was because it was a null result. In the end, we published a whole special issue in BMJ Mental Health on all the key mindfulness trial papers (https://mentalhealth.bmj.com/content/25/3). It’s good that they’re all in one place – over time, it will probably have quite a big impact. But it was disappointing that the big journals still seem to care about the results, rather than the science.

ES: Thinking about your work on adolescence and mental health, is there a paper that you look upon fondly? Do you have a favourite paper, be it the writing process or the actual experimental process?

SJB: Most of my papers I’m fond of, but the one I’m going to tell you about is a review called ‘Development of the Adolescent Brain – Implications for Executive Function and Social Cognition’ (Blakemore and Choudhury, 2006). That is pretty much what I still work on, and it is my most highly cited paper and also one of the most highly cited papers in the journal. I’m choosing it because it was written by Suparna Choudhury, my first PhD student, who I’m still good friends with. We’ve recently written another review paper two decades later (Choudhury et al., 2023). Writing this paper with Suparna and my current PhD student, Blanca Piera Pi-Sunyer, underscores how important the mentoring process is in science and how that relationship can be so productive in both directions. Suparna did her degree in molecular neuroscience whilst I was a psychologist, so I learned a lot from her. She has now moved over to anthropology, so she’s teaching me about another completely different area. Those kinds of mentoring relationships are what I probably enjoy most in my job.

ES: I can comment on mentoring because I’m seeing this here at the Festival – everyone is keen to learn about what everybody else is doing, and to contribute where they can. That kind of process and building up the new generation of neuroscientists is super important. I’m going to go straight to the luxury question – is there something you’d love to have by your side to get you through your writing and publishing process?

SJB: One item that I would love to have is more time to think. It’s difficult to know whether it was always like this in academia, or whether it’s just as you go through different stages of a career – your time to think gets squeezed to almost nothing. I have to ‘diarise’ time to think! Recently, I was writing a paper with members of my group on reinforcement learning in adolescence (Chierchia et al., 2023). The paper went through one review process, and got not so positive reviews. We re-wrote it from scratch, and my PhD student and postdoc came up with a theoretical idea that pulled everything together (about heightened sensitivity and decreased specificity of learning in adolescence). I needed to pencil into my diary a window of time to just sit and think about whether this idea makes sense, working with a pen and paper. That is the kind of luxury that I hardly ever have, and I’m sure lots of people in the audience resonate with that. The luxury of time to think.

ES: So true. If only you had a time machine, hopping back and forth to be able meet people and talk through their ideas. That’s been a wonderful chat with you Sarah-Jayne – thank you so much.

## Shipwreck three


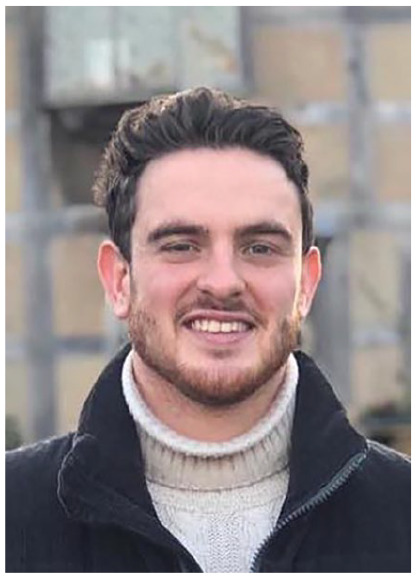
 
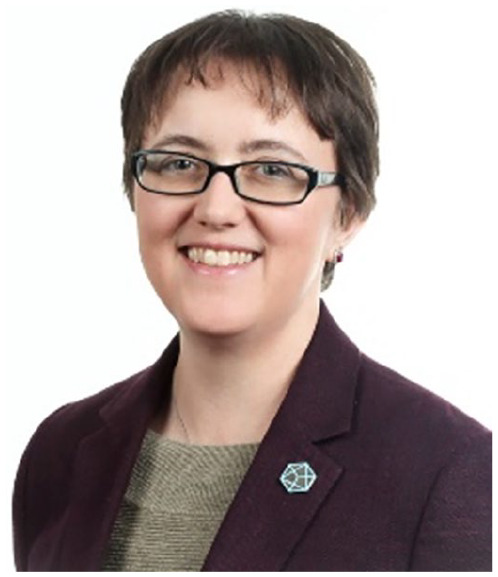


Castaway **Professor Tara Spires-Jones (TSJ)**, Professor of Neurodegeneration and Deputy Director of the Centre for Discovery Brain Sciences at the University of Edinburgh, Group leader in the UK Dementia Research Institute, and current President of the BNA; interviewed by **Dr Danyal Akarca (DA)**, postdoctoral research associate at the MRC Cognition and Brain Sciences Unit, University of Cambridge.

DA: It’s such a pleasure to be chatting with you today, Tara, especially as this is your first day as President of the BNA. To start us off, can you tell us about a paper that you read early in your career that was particularly influential?

TSJ: Absolutely. In fact, I’ll talk about two, because it’s a replication that I still love. During my PhD, which was coincidentally with Sarah-Jayne’s father, Professor Sir Colin Blakemore, I was studying synaptic plasticity and developmental changes in the cortex, but also became interested in neurodegeneration and Huntington’s disease (HD). I fell in love with synapses, and these two papers really pivoted my whole career. Both studies, in two different completely different ways, came to the same conclusion that when you lose synapses in Alzheimer’s disease (AD), your cognitive abilities decline. One paper looked at post-mortem biopsies and electron microscopy – heroic experiments taking tiny samples of human brain and counting individual synapses (DeKosky and Scheff, 1990). The other looked at big paraffin sections stained with synaptophysin and used optical density as a proxy for how many synapses were left (Terry et al., 1991). When I was thinking about what to do for a postdoc, I went to Boston and talked to Brad Hyman, who was working on AD mouse models, and he told me about these two papers, he said ‘You work on synapses, why don’t you work on this?’ – so I pivoted (from HD to AD).

DA: So when you say you pivoted after your PhD, what was that like? You’ve obviously built up a set of skills through your PhD and a set of literature that you’re comfortable with. What was that process like, because I’m roughly at that process now, and I’m sure a lot of people are interested?

TSJ: Mike did this, we heard earlier, and I think a lot of us do. It was kind of scary, but I’d already done it once (I went to do my PhD on developmental synaptic plasticity, and when that project didn’t work I pivoted to work with Tony (Hannan) on the Huntington’s mice and neurodegeneration). They had the synaptic theme, but they were different fields. I still miss the Huntington’s field in a way – the community is amazing. Everybody said ‘You’re going to work on Alzheimer’s disease. Oh, that’s competitive’. But it turns out it’s lovely. So it didn’t frighten me because I’d already done it once and I just loved the synapse – that was the theme that’s run through.

DA: The next question here is ‘what was your first lead author paper?’

TSJ: My first co-authored paper was on electron microscopy of carbon nanotubes (Mitchell et al., 2001). But my first lead author paper in neuroscience was in a society journal, which I’m proud of, and it was on the Huntington’s mouse model (Spires et al., 2004). Tony, Anton and Colin had found that in a mouse model of HD, if you provided environmental enrichment to the mice you slow the progression of the disease (van Dellen et al., 2000). This was not what was predicted – the idea had been that if you overstimulate these mice, you might get more excitotoxicity and make it worse. So when I came into the project, we were really excited to ask ‘what’s the molecular underpinning of this? Why does enrichment actually help and not hinder?’ The project was essentially Western blots and lots of mice. We were looking at BDNF, which is a neurotrophic factor that promotes neuronal survival and synaptic function. We found that the Huntington’s mice didn’t have much BDNF in the striatum. They still had it in the cortex, but it wasn’t being transported to where it needed to be, and enrichment boosted that. It was a great paper, really fun, and I think it was probably one of the most influential papers of my career in terms of impact because it supported the idea that lifestyle changes like exercise and promoting healthy lifestyle can protect you from neurodegenerative diseases. That has really taken off now in Alzheimer’s – about 40% of Alzheimer’s cases could be prevented with lifestyle interventions, including this kind of enrichment (Livingston et al., 2020).

DA: Amazing. What about the people involved? How important was the network of people that you were surrounding yourself with at the time, and how did that shape you? I notice in Cambridge there are so many different types of scientists and I’m trying to learn what kind of scientist I want to be. Could you tell us a little bit about that, maybe?

TSJ: That’s such a big question! One of the people who I admire greatly is Tony (Hannan), who’s here today. His laugh got me through my PhD – you could hear it down the hall, and I really miss that. I was always amazed and am still amazed that Tony seems to have read every paper that’s ever been written, and knows so much about the literature and what’s going on. This was back when we had to actually go to the library and photocopy things, you couldn’t PubMed and find relevant papers. I admire scientists who really understand the literature in depth. I am unfortunately not one of those people because, like Sarah-Jayne, I find every time I want to sit down and read a few papers that my time is sucked away. I also find scientists really inspiring who mentor very well – lots of people that I met at the Huntington’s conferences in my PhD were really influential. Like Mike was saying, people came to my poster and said ‘here’s what I think and I’m helping you along’. That sort of community mentoring is also really important. Finding people you can work with and have great scientific conversations in a casual way where people aren’t too rigid.

DA: I know in our department, we try to foster that kind of open feedback early on, and that’s really wonderful. Next question – what was a paper that was hard to publish? And what aspect of it was hard?

TSJ: I really dithered about whether to do this because I’ve got one that’s still not got a happy ending. It was first submitted in 2019, and there are good and bad aspects of the story. The bad aspect is that it is still not published, and it’s now 2023 – it’s been tough for the lead author and the whole team. Plus, we had the pandemic in the middle, when the mice were all culled. The good aspect is that this paper is by an amazing researcher who did their PhD and postdoc with me (Makis Tzioras). What they did was look in the human brain for the first evidence that synapses might be ingested by glia. This has been shown in mice in some really elegant work by Beth Stevens and Soyen Hong (Hong et al., 2016). We were inspired by that, but mouse brains are in some ways different to human brains, right? Makis first looked in post-mortem human brain and saw synaptic proteins inside microglia. We thought this was amazing, and we submitted it really high (*Science* and *Cell*), and the reviewers were like ‘You’ve got to be joking!’ You’ll find this in whichever part of neuroscience you’re in – there’ll be a few people who are really entrenched on certain ideas, and you have to somehow convince them. Because the reviewers were so harsh and because they gave us some good ideas, we went off and looked at astrocytes, and it turns out the astrocytes are doing more of this (ingesting the synapses) in the human brain than the microglia. We worked with Barry McCall, a microglial expert, to develop a system for growing microglia in a dish and feeding them human synapses. And they eat more AD synapses than control synapses! We expanded that assay to look at astrocytes as well as microglia, and even found a molecular cascade where we can intervene and recover the synaptic ingestion back to control levels. So the paper now is super cool – I love it and it’s out for review again (Tzioras et al., 2023) [Editorial addition: by the time of writing, the paper was finally accepted]. We’ve replicated with lots of different microglial markers and combinations. So it’s solid. I really believe the data and that’s the important thing. Now we’ve talked to a pharmaceutical company who like this cascade and want to explore it more deeply. So in the end, it’s pushed the science forward.

DA: We heard from Sarah-Jayne earlier about slow science and the changing culture around that. Do you think now that people’s perceptions of publishing papers have changed appropriately? Do people really understand that, when looking at a CV, not having ten papers in your early career is normal?

TSJ: I think the whole ecosystem is evolving. Things like narrative CVs help, where you can explain ‘this is a very big project’ or ‘I’ve got data sets’. I personally find a tension in deciding when to stop the project, and when to publish it. Because I want to make sure that everyone who comes through the lab as a PhD student or a postdoc gets a paper out of it. If I waited ten years until we answered all the questions that could harm their careers. So what’s the minimum publishable unit? And a lot of that depends on the people involved in the project and where they are at their career stage, whether we can sit on it for a while or not.

DA: I like the idea of the minimum publishable unit. Derek Jones, in Cardiff, who I collaborate with, calls it the “publicon”. I thought that was a really cool idea. So do you have a favourite paper of yours?

TSJ: This is like my littlest child, who goes ‘I know I’m your favourite, right?’ I thought about it and I’ve picked a paper that we published in 2010 in *Nature* (de Calignon et al., 2010). I’m not picking it because it’s in *Nature*. I’m picking it for two reasons. One is very similar to Sarah-Jayne’s reason – it was the first PhD student that I was properly mentoring. I had spent four years getting the technique to work in mice, to label tangles *in vivo*, to watch them over time. Then I taught this student how to do it – she used the technique that I spent all this effort working on, and it was just wonderful to watch her do it. The other reason I pick it is because, like the enrichment paper, the result was the exact opposite of what we predicted. The prediction was that in AD, you get these big nasty clumps inside neurons called tangles, made of tau. In Alzheimer’s, wherever tangles go in the brain, the neurons die, so we predicted that tangles killed neurons. But nobody had officially shown that, and you never know until you actually look. We had an *in vivo* mouse model that we helped characterise, with lots of tangles and lots of cell death. We make a little cranial window so we could apply stuff to label the tangles, and stuff to show caspase activation (an indicator of apoptotic cells). The prediction was tangle–caspase–dead cell, super easy. But what happened was caspase–tangle–cell survives. We think that soluble tau is building up in that cell and making the cell really sick, then the tau packs itself into fibrils, which are relatively biologically inert, and that actually protects the cell. For as long as we could watch them over time, the cells survived. So that really was paradigm changing in my head and in the wider field as well, that it’s not the tangles, it’s the soluble tau that’s toxic. We actually have a brand-new paper just accepted showing that soluble toxic tau is also the stuff that’s jumping from brain area to brain area through the synaptic connection (Colom-Cadena et al., 2023). So we’ve been building on that idea.

DA: I think it is really interesting how clear your prediction was. It seems to be that really good science necessitates really good predictions. If you get a violation, you really learn something from it.

TSJ: That’s a moral for everybody, right? Don’t be disappointed if your hypothesis isn’t upheld – it’s usually cooler if it isn’t, and you have to do a lot more work to figure-out what’s going on. You have to be bold with ‘what do I think I know?’ and ‘if what I think I know is true, what will I see?’

DA: The next question is what paper or project are you working on at the moment, that you’re most excited about? And we are really interested to know what you think more broadly – what are the questions where we are making headway, or the ones that we should be focusing on?

TSJ: I’m excited as a neurodegenerative disease researcher. For the first ten years I was in the Huntington’s and Alzheimer’s fields, I avoided any meeting with clinical focus like the plague because it was failed clinical trial after failed clinical trial, spending billions and failing to help people, sometimes making people worse. I’m a fundamental neuroscientist studying mice and people’s brains in the lab, but I want this to eventually help people, right? So that was really depressing. But recently as a field, we’re really finding stuff and things are working. In spinal muscular atrophy, which is a horrible motor neuron disease in babies, there’s now an antisense oligonucleotide (ASO) that works (Mercuri et al., 2018). Two days ago, while we were at the Festival, one of the first Phase One papers has come out showing that a tau ASO is safe and it’s lowering tau levels (Mummery et al., 2023). I am not a clinical trials person and I’m not a clinician, but some of the work I did in our lab and as a postdoc was those fundamental biological experiments showing that soluble tau is the stuff that you want to target, not the tangles. I love that we’re finally making a little progress and I think it is a really hopeful time.

DA: Is that due to particular methodological advances or is it more of a theoretical step change?

TSJ: Both. First we were focusing on lesions, those big clumps, and trying to remove them. And that turned out not to be the toxic stuff, but rather the soluble forms are toxic. So that was a conceptual change. But there’s also been huge amounts of progress in the clinical side of things. In the first session of the Festival, James Nicoll gave a talk about one of the first trials for AD to attack amyloid-beta (Nicoll et al., 2019). 30% of the patients in the trial didn’t have AD, which is really going to lower your power to detect whether it was effective, because you’re treating someone with another kind of dementia. So there’s been huge progress in biomarkers and selecting people at the right time in the disease. We’ve also come to the idea that the earlier you treat, the better. So I think there’s been a lot of factors, but what’s beautiful is that it is our whole community, from the fundamental neuroscientists all the way through to the people who characterise the disease in people, and the people who are trying to treat them. It’s a lovely continuum.

DA: It’ll be the last question now about your desert island luxury. One of the PhD students gave the answer of ChatGPT, so maybe we could talk about the implications of that. But unless that is your answer, what would be your desert island luxury?

TSJ: On Desert Island Discs you’re allowed the Bible and Shakespeare, right? So I’m going to presume coffee is already on the island. Then my luxury is a combination of Mike’s and Sarah’s, because when I need to write a grant or a paper, I go somewhere that’s not my house and not my lab. My parents have a tiny beach flat in Musselburgh (on the coast near Edinburgh). I take my dog and my coffee and I go there and write. We also do that for planning – I’ll take a few people when we want to think about something and talk about the idea – what’s the thing that’s important to us? How are we going to do it? I also take my dog – for that love and support, but also that excuse to go outside because he needs to have a walk. So we get a little fresh air and you get some space to think.

DA: Dogs and coffee, that’s given. Thank you, Tara. That was fascinating.

**Top Tips from our Voyagers for Happy and Successful Paper Writing**

- Have something you want to say and know who you are writing for.
- Get feedback at different stages of writing and revising your papers, from people outside your direct research team if possible.
- Use conferences as opportunities to identify areas for further development of a paper, and talk to potential reviewers.
- Carve out dedicated time to write – turn off distractions and make yourself unavailable to interruptions.
- Learn from your role models and enjoy working on papers with mentors and collaborators, but don’t be afraid to develop your own writing style and process which works for you.
- Sometimes it’s a good idea to begin with the Figures, and decide on their sequence.
- Anticipate reviewers’ questions and address these within your manuscript if possible.
- Push back the technical boundaries of the “paper” format: could you make your Figures interactive with embedded data? Could you link to open protocols, datasets and analysis scripts?
- Have confidence in communicating your ideas and your results, even if you are new to an area of neuroscience – no experiment is perfect and future replications will add weight to your findings or refute them.
- Persevere! Papers usually take longer than anticipated to complete and publish, and are often strengthened by the peer review process, although it can be gruelling. Time does not negate the value of publishing your work, and can sometimes increase the impact of your science.
